# A collaborative approach to advancing research and training in Public Health Data Science—challenges, opportunities, and lessons learnt

**DOI:** 10.3389/fpubh.2024.1474947

**Published:** 2024-12-11

**Authors:** Elisha Abade, Wondwossen Mulugeta, Idah Orowe, Getachew Hailemariam, Patrick Weke, Rahel Bekele, Isabelle Zaugg, Jeff Goldsmith, Tiffany Sanchez, Kiros Berhane

**Affiliations:** ^1^Department of Computing & Informatics, University of Nairobi, Nairobi, Kenya; ^2^School of Information Science, Addis Ababa University, Addis Ababa, Ethiopia; ^3^Department of Mathematics, University of Nairobi, Nairobi, Kenya; ^4^Mailman School of Public Health, Columbia University, New York, NY, United States

**Keywords:** public health, data science, DSI-Africa, faculty development, APHREA-DST

## Abstract

The unprecedented availability of increasingly complex, voluminous, and multi-dimensional data as well as the emergence of data science as an evolving field provide ideal opportunities to address the multi-faceted public health challenges faced by low and middle income countries (LMIC), especially those in sub-Saharan Africa. However, there is a severe lack of well-trained data scientists and home-grown educational programs to enable context-specific training. The lack of human capacity and resources for public health data analysis as well as the dire need to use modern technology for better understanding and possible intervention cannot be dealt with currently available educational programs and computing infrastructure, demanding a great deal of collaboration and investments within Africa and with the Global North This paper describes processes undertaken to establish sustainable research training programs and to train a new generation of data scientists with knowledge, mentoring, professional skills, and research immersion. The goal is to position them for rigorous, biomedically grounded and ethically conscious Public Health Data Science practice with a focus on Ethiopia and Kenya. The programs are realized through partnership among Columbia University (CU, USA), Addis Ababa University (AAU, Ethiopia), and the University of Nairobi (UoN, Kenya). In this paper, we describe the collaborative project named “*Advancing Public Health Research in Eastern Africa through Data Science Training (APHREA-DST)”* delving on its conceptualization, implementation framework and activities undertaken. We adopted both qualitative and quantitative approaches to understand the needs of the stakeholders for such educational and training programs. Through harmonized online surveys and stakeholder engagements via focus group discussions in Ethiopia and Kenya, a curriculum was developed for a masters degree program in Public Health Data Science (PHDS). Moreover, the engagement with local projects in both countries as well as active collaboration with other data science related projects in Africa under DSI-Africa consortium benefited the project to start the M. Sc. program successfully. So far, the launching of the graduate program in both countries and the two-cycle experience sharing program done at Columbia University as well as the numerous MoUs signed between partners for data sharing and internships are the major successes of the project. In this paper, we discuss in detail the challenges faced as well as the existing opportunities and lessons learnt this far in implementing this tripartite collaborative teaching and research project.

## 1 Introduction

Over the years, there have been great advances in computing technologies characterized by the emergence of Artificial Intelligence, Internet of Things, Big Data Analytics and Cloud Computing among others. Wide acceptance of these technologies has resulted in increased data processing capabilities in nearly every aspect of modern life. This is catalyzed by increased processing power of modern computing devices.

This technological revolution has resulted in a high volume of data being produced from multiple sources and has propelled the discipline of Data Science which in itself is a multidisciplinary field resulting from the merger of techniques and practices in Computer Science, Statistics and Informatics. Just like Computer Science, application of Data Science cuts across all fields in every sector (1). Some niche areas that have emerged for application of data science techniques include finance, energy, manufacturing, climate (2), environmental sciences and health (2, 3). To meet the growing demand, data science has become a rapidly evolving field of study.

The health sector is one of the priority issues in Sub-Saharan Africa where data science can be highly valuable (4). There are numerous public health concerns in sub-Saharan Africa including Malaria, Cancer, HIV-AIDS, COVID-19 and other regional disease prevalence (5, 6). It is observed that several countries in sub-Saharan Africa are collaborating with a number of development partners in order to put adequate systems in place for effective management of health services. Some of these initiatives have resulted in deployment of Health Information Systems (HIS) which are used in collecting large volumes of data. Such data can be harnessed to bolster data-driven policies and decisions in managing the African health landscape. This often requires sophisticated analyses to be done using Data Science techniques in order to inform the policy makers on appropriate public health interventions and utilization in predicting severity of the diseases. However, there is a severe lack of well-trained data scientists and home-grown educational programs to enable context-specific training.

Although the above mentioned challenges and problems have been well-recognized, previous efforts in improving capacity in the areas of bioinformatics and data science in Africa seem to be limited (7–9). Hence, more effort is still required in this sphere considering the size of the African continent. In order to bridge this gap, a team of researchers from USA, Ethiopia and Kenya proposed to establish a new set of multi-tiered training programs in Public Health Data Science, initially focusing on Ethiopia and Kenya with a possibility of future expansion to other counties in Africa. This new program has been codenamed “*Advancing Public Health Research in Eastern Africa through Data Science Training (APHREA-DST)*” (10).

The APHREA-DST project has been established as a partnership involving Columbia University (CU, USA), Addis Ababa University (AAU, Ethiopia) and the University of Nairobi (UoN, Kenya). It leverages world class strengths in data science at Columbia University (11, 12) to enhance the overall capacity in Ethiopia and Kenya by building upon the readiness and national prominence of both AAU and the UoN. As such the project has developed new context-specific MS programs in Public Health Data Science (PHDS), designed to be sustainable well beyond the funding period of the project. It also includes a faculty mentoring program to build and strengthen capacity in Public Health Data Science for promising Eastern African scientists. Furthermore, it has short-term training programs that are structured around targeted short courses and workshops for a wide spectrum of trainees.

The skills developed through the faculty scholars program are aimed at strengthening the overall training and research capacity in data science in both Ethiopia and Kenya. To broaden the reach into the scientific community, the short-term trainings are designed to engage trainees from partnering governmental and non-governmental stakeholders and the private sector. The program also leverages several ongoing research projects led by team members or affiliated partners on environmental health, exposure assessment, remote satellite data, occupational exposures, climate change, infectious diseases, health surveillance, and health system monitoring and evaluation—ideal as immersion opportunities for trainees to enable hands-on experience with new data science techniques.

With the aim to share experiences related to collaborative program development, this paper outlines the conceptualization and implementation of APHREA-DST, focusing on the collaboration framework, activities, successes, challenges and lessons learnt. The rest of this paper is organized as follows. Section 2 discusses similar works that have been undertaken elsewhere while Section 3 explains the project implementation framework. Section 4 presents specific activities undertaken. Section 5 undertakes a detailed discussion of the training initiatives, program sustainability, skills transfer, challenges, opportunities, and lessons learnt in the course of project implementation. Finally, Section 6 provides conclusions and recommendations for future directions. The document ends with an acknowledgment of the funding received from the United States National Institute of Health that has enabled the implementation of this project.

## 2 Related works

There has been a growing interest in the use of data to generate insights and solve health problems in Africa. However, it is now widely recognized that there is a gap in technical know-how required to take advantage of the increasing abundance of data in solving African health issues. In recognition of this, there are initiatives similar to APHREA-DST that are currently ongoing in Africa. For instance, SSACAB project (7) is among the pioneer collaborative initiatives aimed at developing capacity of African researchers and professionals in Biostatistics, which is a very important component in Public Health Data Science. A team of researchers in Ethiopia (8) in their study, have presented a roadmap for building capacity for health discovery in Africa, providing some initiatives including capacity building of academic staff in African universities. Additionally, in West Africa (9) there is an ongoing effort to outline ways of building capacity through training in Bioinformatics and Data Science.

Within the DS-I Africa initiative (13), there are also a number of projects focusing on training data scientists for Africa and thus promoting the use of data in decision making while solving the challenges in African health landscape—including APHREA-DST discussed in detail below.

## 3 Project framework for APHREA-DST

APHREA-DST is one of the training programs in the larger Data Science for Health Discovery and Innovation in Africa (DS-I Africa) (13) consortium of projects being funded by the Common Fund of the US National Institute for Health (NIH) (14). The DS-I Africa initiative aims to leverage data science technologies to transform biomedical and behavioral research and develop solutions that would lead to improved health for individuals and populations. It also aims to create and support a robust pan-continental network of data scientists and technologies that will be equipped to apply advanced data science skills to transform health.

**Figure 1:**
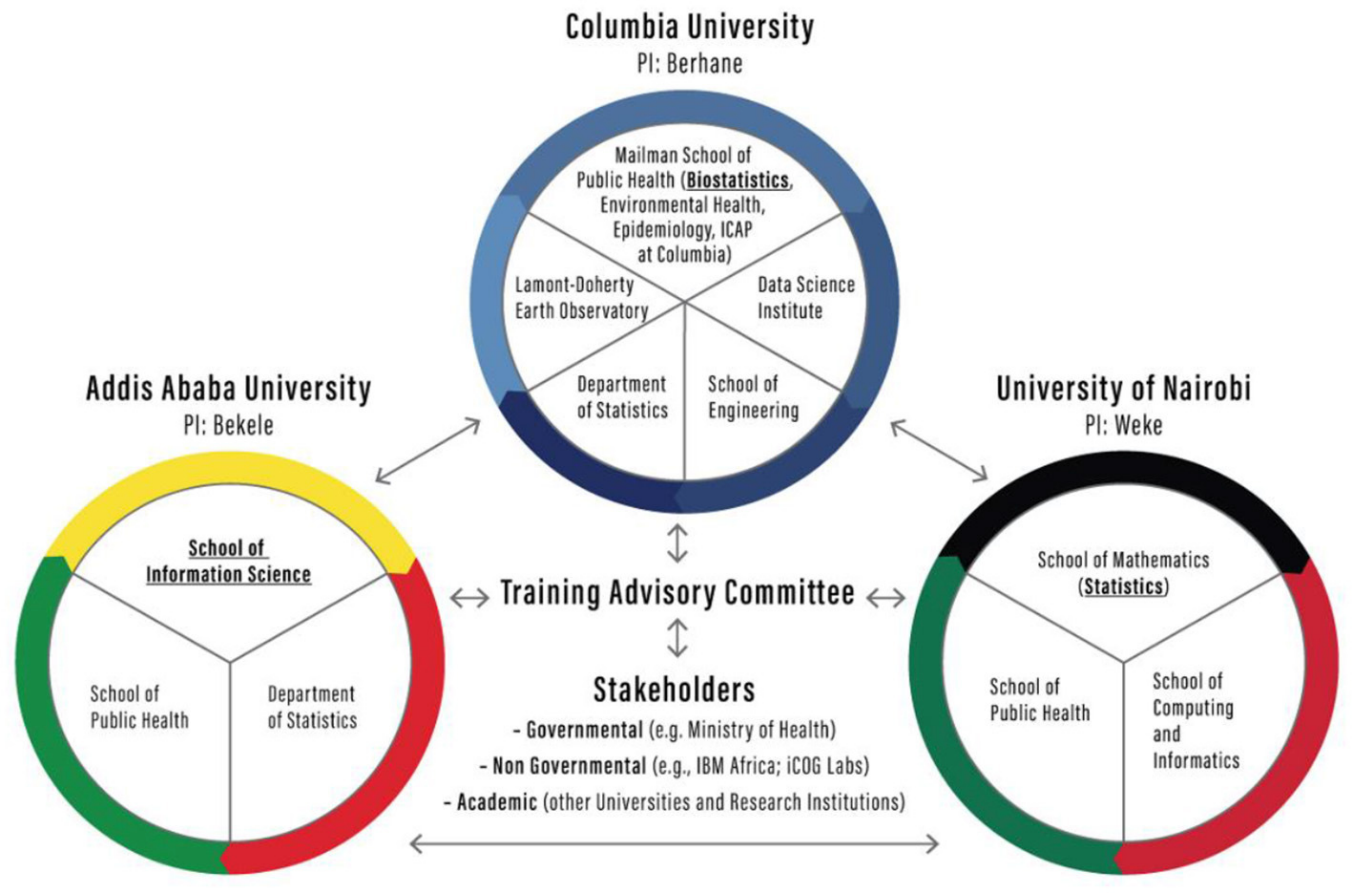
Institutions collaborating in the APHREA-DST project.

Being a tri-parte project comprising researchers drawn from universities in the USA, Ethiopia and Kenya, each collaborating partner has a country team led by a Principal Investigator (PI). APHREA-DST assembled multidisciplinary team of researchers drawn from multiple departments in each of the participating universities (Figure 1).

**Figure 2:**
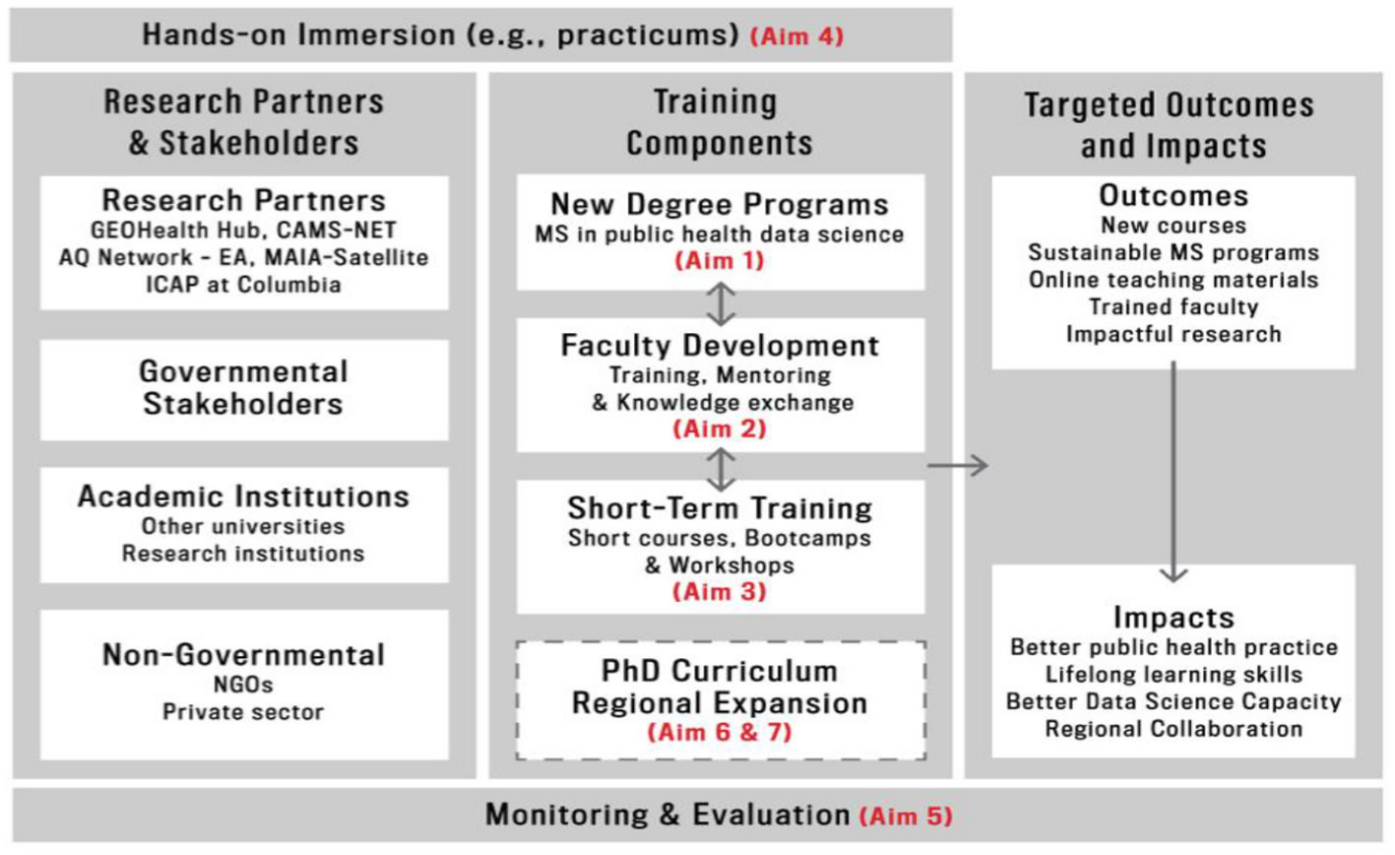
The project framework.

The overall organization of the APHREA-DST project envisions tight collaboration with research partners and stakeholders, a mix of existing research initiatives in the partner institutions as well as governmental, non-governmental and academic partners. The collaboration feeds into the overall objective of building capacity in data science through establishing a new masters graduate program, faculty development and short-term training (Figure 2). Further, the targeted outcome and impacts of APHREA-DST are new, sustainable graduate program and better public health practices.

**Figure 3:**
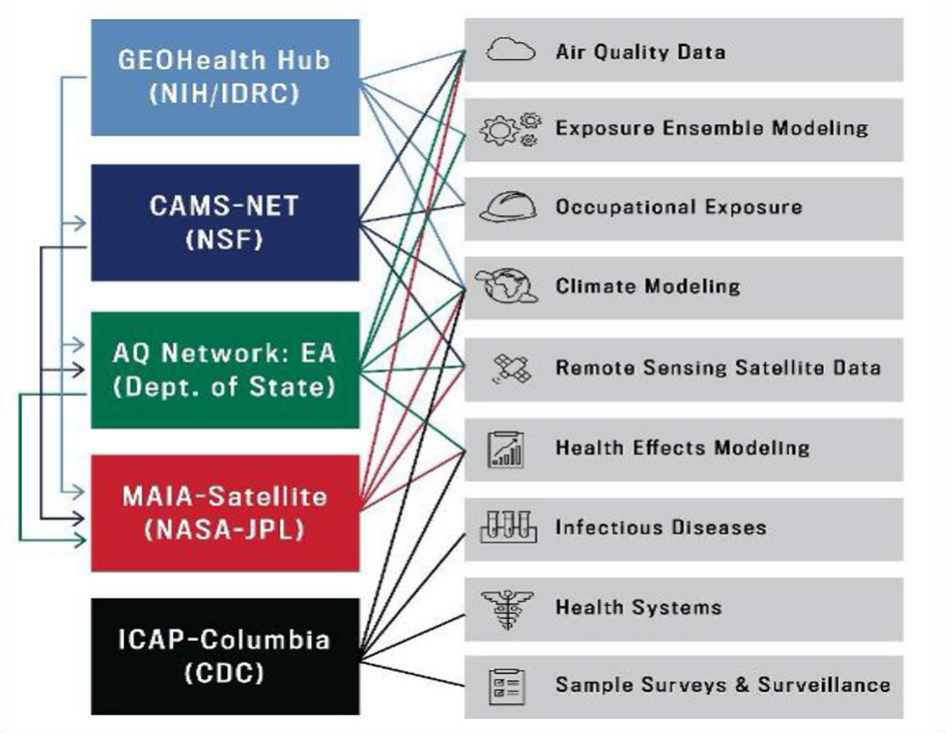
The APHREA-DST data partners.

Other than the three universities, APHREA-DST has been joined by partners including several research oriented research hubs and research oriented organizations so that they can play various roles including provision of data that will be used in the course of collaboration. Major partners include GEOHealth Hub (15), CAMS-NET, AQ Networks, MAIA satellite, and ICAP-Columbia. Figure 3 summarizes the types of data that are available within the projects.

## 4 Project activities

APHREA-DST was conceptualized to comprise a series of interconnected activities to be conducted at various carefully structured time intervals. This section highlights some of the activities that have been undertaken so far and the associated experiences.

### 4.1 Needs assessment

The need for data science in the health sector is well-noted by many renowned universities all over the world. In line with this, the need for graduate programs, training and research in health data science is well-acknowledged. Additionally, paying closer attention to the local context in order to address the need for health data science training is important. With this rationale, APHREA-DST researchers undertook a needs assessment study with relevant stakeholders to understand the real need for graduate programs, related curricula as well as short term training in health data science. The needs assessment was also important in:

i. Finding best approaches for shaping the curriculum,
ii. Identifying relevant topics that would have greater impact in the local context,
iii. Identifying potential candidates for the master's degree program,
iv. Deciding on the most conducive approach to delivery of the training program(s), and
v. Identifying research and short-term training topics.

Data collection was conducted using an online platform and the respondents for the survey were identified in collaboration with the stakeholder's liaison team. The data collection instruments were designed to explore and quantify demographics, work experience, skills and interest areas, need for graduate level training, thematic research areas and short term training needs. The respondents ranged from practitioners in the areas of health sciences including public health practitioners and medical doctors to ICT experts and academicians.

Most importantly, the study focused on looking into the skills and interest areas from five categories, namely: data proficiency, statistics, advanced modeling, public health, and knowledge synthesis with focus on current and desired skill levels.

**Figure 4:**
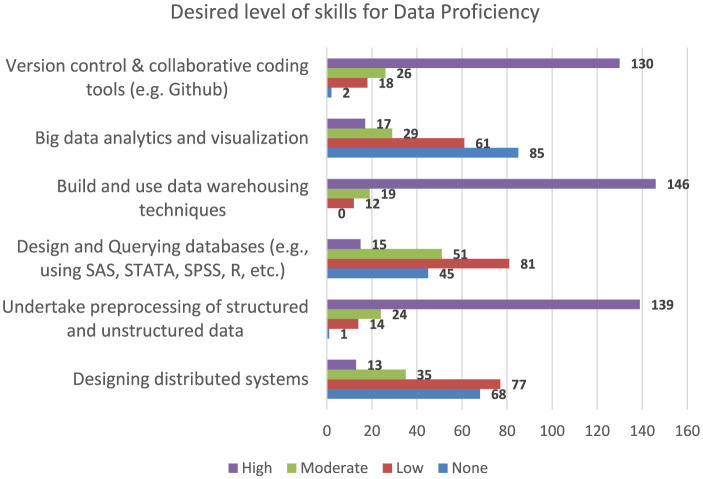
Desired level of skills for data proficiency (Ethiopia).

The needs assessment survey had a good response rate and acceptable representation from various related fields of studies as well as demographic parameters. While each parameter was well-analyzed, the response from the respondents showed that there is a great need for a Public Health Data Science program with identified focus areas. Figure 4 illustrates one of the infographics that showed the need for data proficiency skills by respondents in Ethiopia. It can be inferred that the respondents expressed high level of interest in gaining skills in data warehousing techniques, processing of structured and unstructured data as well as use of version control and coding tools.

**Figure 5:**
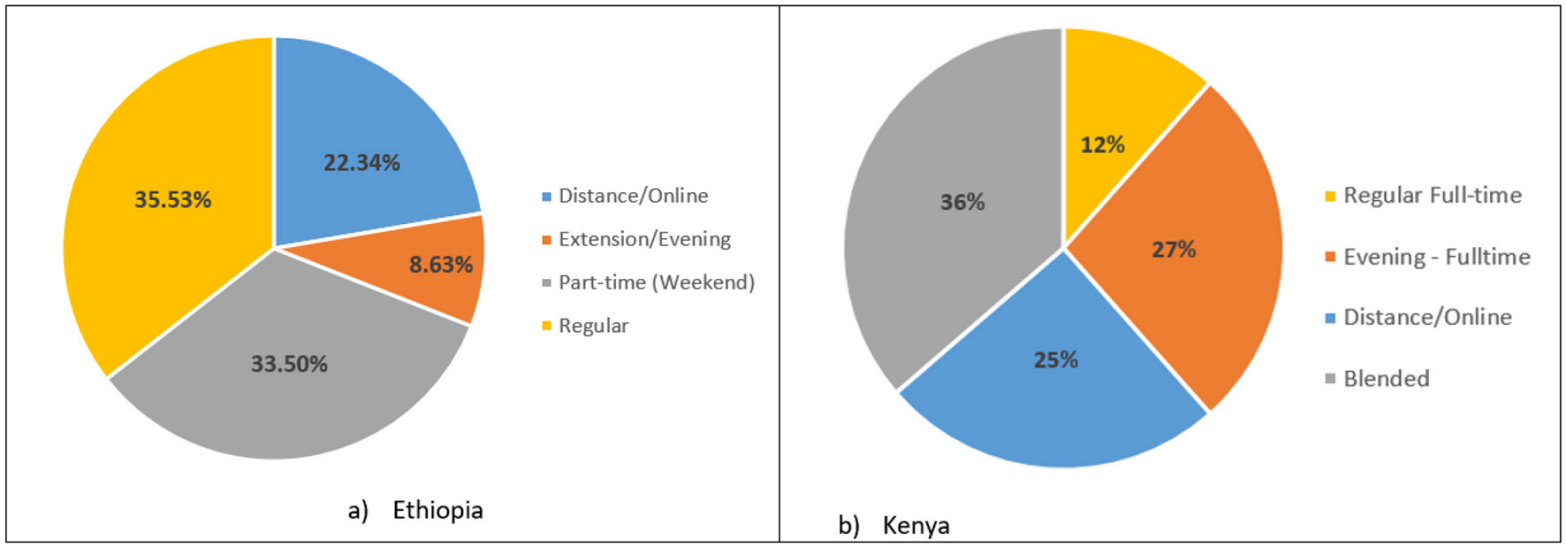
Preferred mode of delivery for the MS program. **(a)** Ethiopia. **(b)** Kenya.

The assessment also sought to establish preferences of the prospective students with regards to modalities of course delivery. As shown in Figure 5, in Ethiopia, regular and evening delivery are the top two preferred modalities while most Kenyan respondents preferred either blended learning or part time (evening) classes as modalities for delivering the proposed course. These findings formed the basis for the design and implementation of the program. The new master's degree program was launched at the UoN in January 2023 and at AAU in Fall 2023.

With regards to research topics, public health data tracking and analysis and predictive analytics for pandemics emerged as top priority areas for both countries as shown in Table 1. Other areas of relatively greater interest were monitoring and evaluation of public health data, time series data analysis and public health surveillance and survey.

**Table 1:** Top 3 areas for research in each country.

**No**	**Ethiopia**	**Kenya**
1	Public Health Data Tracking and Analysis	Public Health Data Tracking and Analysis
2	Monitoring and Evaluation for Public Health Programs	Predictive Analysis for Pandemics
3	Public Health Surveillance and Survey	Time Series Data Analysis

**Figure 6:**
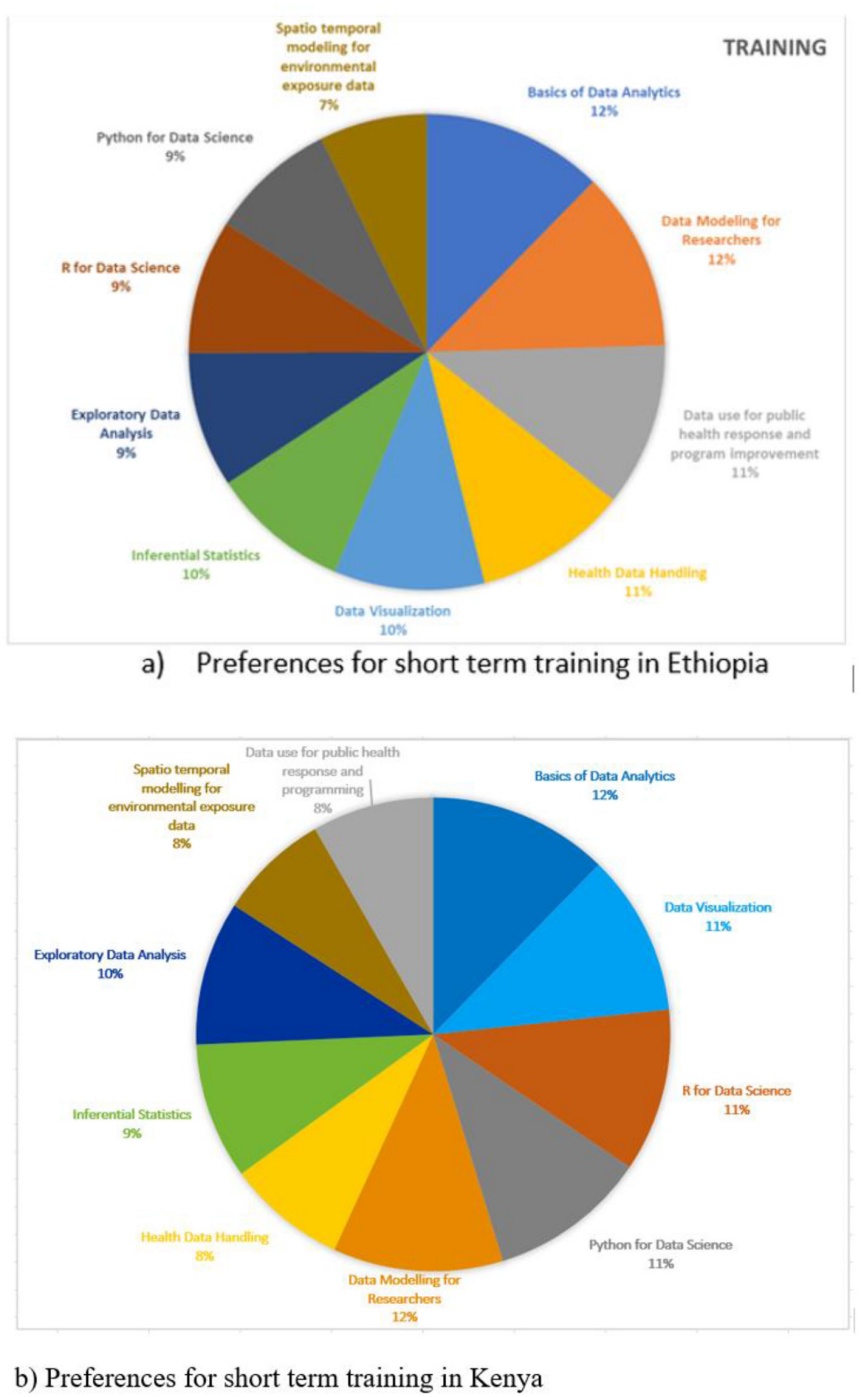
**(a)** Preferences for short term training in Ethiopia. **(b)** Preferences for short term training in Kenya.

Capacity building can also be achieved through short term training on selected topics. The needs assessment survey indicated that training topics on Basic Data Analytics, Data Modeling for Researchers, Data use for Public Health, and Health Data Handling were ranked top by majority of the respondents in both Ethiopia and Kenya. These were closely followed by Data Visualization and Inferential Statistics as illustrated in Figure 6.

### 4.2 Stakeholder engagement

APHREA-DST subscribes to project management best practice requirements (16) of engaging stakeholders at all stages of a project, right from inception. The project team actually began by mapping out all possible stakeholders in establishing a university-level training curriculum. The stakeholders were drawn from a wide range of institutions and individuals, including potential students, prospective employers, data partners, government agencies, and regulators in the health sector, among others. This does not only strengthen the public participation requirement but also ensures that the needs of these stakeholders are taken care of while developing the curriculum. It was also noted that the potential students were from a wide range of academic backgrounds including Mathematics, Medicine, Computer Science, and Biostatics among others.

The engagement with stakeholders confirmed that key influential partners (both from government and not government organizations) considered in the study have strong support and desire for data science training programs. Even though limited knowledge and understanding was observed in a few study settings regarding data science, the overwhelming majority of stakeholders are well aware of the potential benefits of data science in public health and in the health sector at large. Unprecedented willingness for data sharing, collaborations, and engagement have been witnessed with all stakeholders. In fact, most of the stakeholders have clearly articulated their needs surrounding data science expertise and about the prevailing skill gaps, short term training needs, and potential collaborations in training and research engagements. Further, they have expressed their commitment and willingness for data sharing.

In general, the ongoing APHREA-DST project has gained a wider acceptance and recognition from the academic community, public health practitioners, research community, and top management of both AAU and UoN.

### 4.3 Curriculum development

One of the objectives of the APHREA-DST project is to revolutionize data science training in Africa through establishing a Master of Science degree program in Public Health Data Science in both Ethiopia and Kenya. To this effect, the project embarked on a curriculum development initiative aimed at creating a new curriculum for teaching Public Health Data Science at master's level at both AAU and UoN. The curriculum development endeavor was primarily guided by the outcome of the need assessments, stakeholder engagement activities and best practices drawn from all the three participating universities, that is, CU, AAU, and the UoN.

Therefore, a curriculum development team comprising all stakeholders from CU, AAU, and UoN was formed. The team took charge of developing the curriculum borrowing from the experience of CU. This was a rigorous process that entailed several team meetings and workshops facilitated by pedagogy experts. The curriculum development team also had to put into consideration the fact that AAU and UoN have their own distinct institutional requirements for academic curricula and their respective in-country regulatory obligations (17, 18).

The topics covered in the curricula are drawn from three disciplines, namely: Computer Science, Statistics and Public Health. The contents of courses drawn from Computer Science and Statistics and their corresponding learning outcomes have been mapped onto the Association for Computing Machinery (ACM) 2023 Computer Science curriculum in terms of developing analytical and problem solving skills, knowledge of algorithms and data structures, strong mathematical and logical skills and ethical behavior. The contents map onto the following Bodies of Knowledge as proposed in the ACM 2023 Computer Science curriculum: Artificial Intelligence (AI), Data Management (DM), Networking and Communication (NC), Mathematical and Statistical Foundations (MSF), and Society, Ethics and Professionalism (SEP) (19).

The contents in public health include course units in Epidemiological Methods; Health Planning, Management and Policy Development; Public Health Data Engineering and Management as well as Health Data Mining and Analytics. It is expected that upon successfully going through the proposed curricular, the graduates should not only be able to harness the massive public health data in Africa to proposed conducive public health interventions but also be able to:

i. Integrate components of data analytics to produce knowledge-based solutions for practical challenges using public and private data sources.
ii. Identify determinants of health and disease, public health practices, health service structure as well as health data policy and strategy.
iii. Apply a variety of Machine Learning algorithms and techniques to analyze public health data;
iv. Perform innovative, novel, and applied research in data science;
v. Resolve ethical and legal issues relevant to a data analytics or a research project.

The results of this activity are curricula that not only conform to respective country requirements in both Ethiopia and Kenya but also have striking similarity in key areas targeted for capacity building allowing for synergy and collaboration. The courses are listed semester-wise as shown in Table 2. A detailed listing of the contents of these courses is separately accessible from the website of the UoN's department of Mathematics.

**Table 2:** MS Public Health Data Science curricula in Ethiopia and Kenya.

**a) AAU curriculum**		**b) UoN curriculum**	
**First semester**	**Second semester**	**First semester**	**Second semester**
**Year 1**			
• Epidemiology • Biostatistical Methods I • Data Science Fundamentals • Statistical Computing	• Probability and Statistical Inference • Machine Learning • Research Methods for Data Science • Public Health Data Engineering and Management	• Epidemiological Methods I • Biostatistical Methods I • Data Science Fundamentals • Statistical Computing	• Probability and Statistical Inference • Machine Learning • Biostatistical Methods II • Epidemiological Methods II
**Practicum/internship**			
**Year 2**			
• Advanced Data Analytics • Biostatistical methods II	• Thesis^a^	• Health Data Mining and Analytics • Advanced Machine Learning • Research Methods in Data Science	• Research Project in Public Health Data Science
		**Electives**	
		• Big Data Infrastructure, Platforms and Warehousing • Data Security and Privacy • Machine Vision for Data Science • Internet of Things • Longitudinal Data Analysis • Survival Data Analysis • Health Planning, Management and Policy Development	

^a^Equivalent to the UoN research project in Public Health Data Science.

### 4.4 Project launch and training initiatives

Following the planning and preparatory activities, the APHREA-DST project was launched in 2022 and several training activities have been undertaken to date. The subsequent sub-sections give details into these initiatives.

#### 4.4.1 Project launch

The APHREA-DST project was officially launched in Nairobi, Kenya in a weeklong set of activities between 4th and 8th April, 2022. The activities included engagements with senior management of the UoN including the Vice-Chancellor, his deputies and other stakeholders. During the launch, experts from CU conducted a faculty capacity building training on various topics to enhance the capability of faculty from both AAU and UoN.

#### 4.4.2 Training initiatives

Training and capacity building is one of the key pillars of the APHREA-DST project. This is structured into two broad classes, namely the formal academic training at MS level and short-term trainings lasting variedly from 3 days to several weeks. A key deliverable of the project remains the launching a new Master of Science (MS) programs in Public Health Data Science at AAU and the UoN. To this effect, the UoN started admitting students in later 2022 to January 2023 as the first cohort of MS Public Health Data Science students. This cohort comprised 15 students, 11 of whom graduated in September 2024. Moreover, UoN has continued to admit additional two cohorts of students in 2023 and 2024, respectively.

At the UoN, delivery of the courses offered in this MS program has heralded a new dawn of multidisciplinary teaching involving faculty from all the three participating departments. Each department leverages their expertise to ensure that the students get the best. Further, the members of faculty who have gone through the faculty development program have been of immense benefit to the UoN when it comes to skills transfer. All the four scholars have not only delivered the courses but have also utilized some of the learning resources and case studies that they acquired from their mentors, most of whom are from CU. Similarly, AAU admitted fifty students in September 2023. AAU is currently accepting applications to recruit and admit second-cohort students into the regular and extension programs.

The short-term training in data science and machine learning has been conducted for both project staff and stakeholders. Over the past 3 years several short courses, bootcamps and workshops have been conducted. These short-term trainings and bootcamps have been structure to cover key areas that were found out after the needs assessment and stakeholder engagement exercises. Some of the topics covered include: high-dimensional data analyses, Building Shiny apps using R, Data storage and retrieval using SQL, Python programming, and general concepts of Machine Learning as well as advanced topics in Deep Learning. The short term trainings have been conducted in the US at Mailman School of Public Health as well as in Ethiopia and Kenya during the annual spring workshops.

### 4.5 Faculty development

The faculty development program was started primarily by engaging the faculty members of AAU and UoN, working with mentors from CU, in the overall project administration and joint project engagements. Faculty scholars were made to play a major role in needs assessment, stakeholders' engagement and most importantly in curriculum development activities. These activities allowed the faculty members to interact and co-work with senior professors and expand their knowledge and skill in various academic activities.

The faculty development program is run on annual basis whereby each year, a different cohort of academic staff from UoN and AAU is enrolled into the program. As such, each year, two faculty from each university are selected as faculty scholars. The faculty scholarship entails a rigorous set of activities throughout a full-year period including undertaking regular meetings between a CU based mentor (working with a pair of mentees—one each from Ethiopia and Kenya), targeted short term training (e.g., bootcamps on selected topics) and a week-long visit to CU for intensive training and immersion with various CU based centers and schools.

### 4.6 Research groups

As shown in Table 3, thematic Research Groups (RGs) have been established in both Ethiopia and Kenya to streamline the teaching and research undertakings of the program. Instructors and students join the research group based on their interest and expertise and in line with the course they teach in the program. Students are expected to be involved in the RGs after completion of their 1st-year studies. Core members of the APHREA-DST project as well as other instructors assigned to teach and advice students in the program are expected to be members of the RG. The duties and responsibilities of the Research Groups include:

i. Identifying potential research areas.
ii. Suggesting instructors that will teach courses.
iii. Proposing teaching strategies.
iv. Mentoring students in selection of topic and drafting a research proposal.
v. Reviewing and approving the research proposals.
vi. Suggesting research advisors to students.
vii. Identifying short-term training needs.

**Table 3:** In-country research groups.

**AAU Research Groups**	**UoN Research Groups**
• Data warehouse architecture • Maternal and Child Health • Communicable & Non-Communicable Disease • Environmental health	• Statistical and Predictive Modeling • Machine Learning and Data Visualization • Big Data Analytics • Data Security Privacy and Ethics • Genomics

The Master's programs in Public Health Data Science at both AAU and UoN are supposed to pursue similar goals and objectives but at the same time pay attention to priorities and unique opportunities in each country. Accordingly, both the UoN and AAU teams have identified the following Research Groups.

Although slight variations are observed in the naming of the Research Groups, there is still a high degree of overlap in areas of interests which are also motivated by in-country priority areas and profiles of the team members representing both AAU and UoN. These Research Groups are still expected to be the main drivers of the teaching and research programs at both ends.

### 4.7 Monitoring and evaluation

The project has adopted a Monitoring and Evaluation approach that follows a logic model, moving from proximal and immediate indicators, such as recruitment of trainees and their success in meeting competency-related Individual Development Plan (IDP) goals and conducting practicum research, to long-term indicators such as implementation of sustainable Public Health Data Science programs and the ability to conduct data-science-related research. The project aims at ultimately improving regional public health outcomes through development of a well-trained and public-health-focused generation of data scientists. The sub-section that follows gives details of the monitoring and evaluation approaches that have been adopted in the project.

#### 4.7.1 The monitoring and evaluation process

For the MS programs, each university already has well-established rules and regulations governing implementation of the courses as well as country-specific Quality Assurance mechanisms. In order, to meet compliance requirements, the APHREA-DST project tapped into these established in-country Quality Assurance mechanisms with a view to assessing the quality and effectiveness of the trainings. In the case of MS programs, these approaches include course evaluation through obtaining feedback from students at the end of every semester so as to inform future enhancements and surveys upon graduation. For the short courses, the adopted approaches include pre- and post- course surveys. The summary of such findings are discussed with the Teaching Advisory Committee (TAC). In general, the evaluation methods, with some variation depending on whether it is for the MS program or for the short courses as well the country of domicile, include:

i. Document review (such as admission rates, percentage of trainees from disadvantaged or underrepresented groups in the program such as number of women, those with disabilities and graduation rates).
ii. Surveys of participating trainees at baseline, following completion of specific program activities, and annually thereafter (especially for the faculty scholars);
iii. Surveys of mentors; and
iv. Interviews with trainees, mentors, faculty, and the TAC.

Since the inception of the program, APHREA-DST has impacted 164 MS program students in both Ethiopia and Kenya, 50 at AAU and 114 from the UoN. Of the 114 students enrolled in the MS program, the first cohort had only 15 students, 11 of whom have already graduated and two of them have been retained by the UoN for part-time teaching as assistant lecturers.

Most of the trainees for the short term programs are employees already working for various corporates in Ethiopia and Kenya. Similarly most of the students enrolled in the MS program are already employed and they have been retained by their employers both during the course and after graduation. The ones who were working before continue to work in their respective organizations. So far, only two students were forced to terminate their employment in order to pursue the masters programs. Further of the 114 students from the UoN, 42 are women while 72 are men, hence women account for 36.8% of the UoN students. On the other hand, of the 50 students from AAU, 48 are men while only two are women. The AAU has outlined a deliberate policy to bridge the gender gap in subsequent admission cycles. Such policies include deliberate advocacy initiatives to onboard female students in the project.

#### 4.7.2 Long-term and regional impacts

The direct long-term impact of APHREA-DST project is 2-fold, namely:

i. To bridge the Data Science skills gap in Africa, especially with regards to Public Health Data Science.
ii. To improve regional public health outcomes through enhancing data driven interventions in public health and development of a well-trained and public-health-focused generation of data scientists.

Therefore, beyond the educational programs and collaborations, our project is designed to cultivate long-term regional collaboration, lifelong learning skills, and a supportive community of researchers committed to open science, algorithmic fairness, and “data science for good,” ultimately leading to better public health practice. Ultimately, in the 5th year of the project, we will broaden the training program to the wider East Africa region through sharing of curricula and inviting trainees for engagement. We will also explore the feasibility of incorporating the courses we have developed into existing PhD curricula or creating new Ph. D. programs in Public Health Data Science.

## 5 Discussion

Since the official launch in Nairobi in April 2022, APHREA-DST has undertaken several activities as described in Section 4 above. Among the key accomplishments are the successful development of a curricula for master's in Public Health Data Science and faculty scholar development initiatives. In order to ensure that the program runs smoothly, beyond the funding window, the implementation team has put in measures to ensure sustainability, some of which are outlined in the section that follows.

### 5.1 Sustainability

Sustainability is core to the project goals. This has been closely intertwined with the core components of the project. Such measures include:

i. Faculty development implemented through the faculty scholars program. This initiative provides for faculty from both AAU and UoN to be mentored by faculty from CU in order to strengthen and improve their data science capacity. These faculty scholars end up forming the core of Data Science experts running the APHREA-DST training programs in their respective countries. Every year, there are two faculty scholars nominated from each country to be mentored at CU through this initiative. Currently, in each country there are four faculty scholars who have already undergone through this program and the next cohort of two scholars from each country have just been selected. They will undergo 1 year faculty development program.
ii. Retention of some of the students who have been trained in the MS program as assistant tutors and with a view to having them pursue PhD degrees so as to allow them to join full-time faculty thereby boosting the existing local capacity in Public Health Data Science.
iii. Admission of self-sponsored students to ensure that we will still have students even when there are no scholarship opportunities.
iv. Short-term trainings will serve as a vehicle to build ties with key stakeholders namely: trainees from other universities in the respective countries (Kenya and Ethiopia), experts in governmental agencies (such as Ministry of Health), ICT companies in the private sector and non-governmental agencies with objectives of expanding and diversifying the STEM workforce.
v. Many of these stakeholders will also partner with our program on research projects that will provide immersion, internship, or work-study experiences within their organizations for our students hence offering avenues for continued sustainability of the program.

### 5.2 Skills transfer

One of the anticipated long-term impacts of this project is to bridge the Data Science skills gap currently being exhibited in several African countries. Beyond the MS program being run at AAu and the UoN, APHREA-DST project has put in place a number of skills transfer initiatives aimed achieving the objective of bridging this skills gap in Data Science. These include:

i. The faculty development program in which members of faculty from East Africa are paired with their peers at Columbia University for enhanced capacity building in Data Science as elaborated in the previous sub-section.
ii. Short-courses in which a number of corporate clients send their members of staff for training in Data Science at both AAU and UoN hence the members of faculty help in skills transfer to the industry task-force.
iii. Workshops: The project conducts annual workshops every spring season where the participants show-case their Data Science works and also short-term trainings are conducted hence the workshops form core or our skills transfer avenues.
iv. The DS-I Africa working groups: The APHREA-DST is one of the projects under the consortium known as Data Science for Health Discovery and Innovation in Africa (DS-I Africa) Initiative (13). The consortium has very vibrant working groups which meet biweekly to discuss thematic issues in several areas of Data Science including data management. They thus form the core of the skills transfer initiatives availed within this project.

In the course of implementation of this project, the team has faced a number of challenges and also identified several opportunities that can be harnessed to achieve the intended objectives. In the process, we have also learnt a number of lessons with regards to collaborative curriculum development. In subsequent sections, we discuss some of these.

### 5.3 Challenges

Managing a project of such a magnitude as APHREA-DST is inherently laced with challenges that project managers and key team members must be prepared to overcome. A key challenge in the earlier stages of this project was developing modalities for buy-in by key stakeholders. For instance, APHREA-DST was launched at the time when some partner institutions were undertaking financial, structural and human resources reforms. Introducing a new curriculum in such an environment that was characterized by archiving and sun-setting of existing programs that were perceived as either unpopular or “non-performing” was not an easy task.

Additionally, APHREA-DST is inherently a multi-stakeholder project. While balancing the interests of all stakeholders is already a daunting task, the jurisdictional differences meant even a more difficult scenario for the project managers, especially the different regulatory requirements faced by each of the participating universities. Further, both AAU and UoN have different procedures for curriculum development and approval. The bureaucratic nature in some of the curriculum approval stages resulted in time overruns thus impacting the schedule of program launch—which fortunately amounted only to a delay of a few months.

### 5.4 Opportunities

Despite the challenges enumerated above, APHREA-DST is also presented with several opportunities that the project team is keen on exploiting. First and foremost, in both Kenya and Ethiopia, the governments have forged close working relationships with the development partners in enhancing service delivery in the health sector. The governments in both Ethiopia and Kenya have rolled out country-wide Health Information Systems, specifically District Health Information System (DHIS2) (20, 21) which is used for collection of health data hinged on a number of health indicators. DHIS2 is used to collect aggregated data at the facility level and transmitted to the national level paying keen attention to geographic and administrative levels in each country. The massive amount of data generated and processed in DHIS2 makes it an obvious source of data for use by Research Groups.

Both Ethiopia and Kenya face nearly similar public health challenges and are thus in need of improved public health services. It is noteworthy that the two participating universities in both countries have well-established undergraduate programs in health sciences, Mathematics, Statistics and Computer Science hence the students graduating from these programs will have a good background to pursue advanced courses in data science such as the newly launched MSc. Program in Public Health Data Science.

Another opportunity that APHREA-DST must ride on is the well-established collaboration and knowledge exchange initiatives including the partner universities within the larger DS-I Africa initiative, the AI-Data Science and Theoretical and Computational Thinking Initiative and African Engineering and Technology Network (Afretec) (22). Such networks offer a conducive ground from which APHREA-DST can access not only human resources but also data that are key ingredients for its success.

The APHREA-DST needs to solidify and legitimize the existing motivation and interest for collaboration through signing of Memorandum of Understanding with these stakeholders, especially for data sharing and offering of practicum and internship opportunities for students of the graduate program in Public Health Data Science. Attention must be given to data sharing agreements owing to institutional policies and national legislations such as the Data Protection Act 2019 in Kenya (23) that govern data sharing. While formulating the engagement strategy, it is also imperative to note that stakeholders need to be classified based on their level of influence and support.

Following this, a corresponding strategy needs to be formulated to guide on issues such as, with whom to build high level collaboration and engagement, who should be consulted at key decision points, who should be simply informed about the progress of the program and who deserves and requires strong ties and work relationships.

### 5.5 Lessons learnt

In the course of implementing key components of the APHREA-DST project, there have been a number of lessons to be learnt by the implementation team. First, it became clear that for such a multi-stakeholder project, it is critical to engage as many stakeholders as possible and as early as the inception phase of the project. This is especially true for the data partners since there are several institutional policies that affect data sharing.

It was also learnt that in order to ensure that activities are undertaken in accordance with the work plan, project implementation team must be ready to hold constant and regular team meeting where progress is evaluated and any bottlenecks that impede progress are solved in a timely fashion. This calls for serious and total commitment by all team members.

Another lesson learnt is that it is important to identify the prevailing jurisdictional restrictions and regulatory requirements when implementing a project in a highly regulated sector like higher education. Further, it is of great essence to take into considerations the timelines required by various approval steps, the prevailing institutional dynamics notwithstanding.

## 6 Conclusion

The experience with APHREA-DST has revealed that the demand for data science transcends the health sector. The project may, thus, serve as a springboard for opening more generic data science programs at undergraduate and graduate levels. Therefore, the partner universities need to consider generic data science training programs to further sustain the existing initiatives and to meet the growing needs. The project has also uncovered some innovative initiatives and tech savvy entities and hubs such as the GEOHealth hub, Afretec, and AI-Data Science and Theoretical & Computational Thinking Initiative.

These initiatives can be good sources of data and data science expertise for the APHREA-DST project. Therefore, building strong partnership with such entities would strengthen and facilitate the project undertakings.
